# Molecular Evidence of *Theileria* and *Anaplasma* Species in *Hyalomma marginatum* Ticks Infesting Goats in Qinghai, China

**DOI:** 10.3390/vetsci13101078

**Published:** 2026-10-12

**Authors:** Peiyao Yang, Lamu Aan, Chengcai Wang, Yi Yang, Zili Dime, Xuepeng Liang, Zhi Li, Dan Jia, Xueyong Zhang, Xiuying Shen, Yong Fu, Yijuan Fu, Jie Wang, Hong Duo

**Affiliations:** 1 Academy of Animal Sciences and Veterinary Science, Qinghai University, Xining 810016, China; 2 Key Laboratory of Animal Disease Pathogen Diagnosis and Green Prevention and Control Technology of Qinghai Province, Qinghai University, Xining 810016, China; 3 Qinghai Provincial Animal Disease Prevention and Control Center, Xining 810001, China; 4 Department of Animal Science, Qinghai Agri-Animal Husbandry Vocational College, Xining 812100, China

**Keywords:** *Hyalomma marginatum*, Qinghai Province, *Theileria*, *Anaplasma*

## Abstract

Ticks are obligate hematophagous arthropods and important vectors of various pathogens. This study identified tick species parasitizing goats in Guide County, Qinghai Province, China, and screened for *Theileria* and *Anaplasma* species. Tick specimens were identified via morphological observation and molecular analysis of *16S* rRNA and *cox1* gene sequences. Pathogen detection was performed using PCR targeting the *18S* rRNA gene of *Theileria* and the *groEL* gene of *Anaplasma*, followed by sequencing and phylogenetic analysis. Morphological and molecular data consistently identified all 80 engorged tick samples collected from goats on a single farm as *Hyalomma marginatum*, with 97.7–99.7% sequence similarity to reference sequences. Among the 80 tick samples, the DNA detection rate for *Theileria ovis* was 5.0% (4/80), 17.5% for *Theileria luwenshuni* (14/80), and 6.3% for *Anaplasma ovis* (5/80). In this study, *H. marginatum* was identified in Guide County, Qinghai Province, thereby enriching the current knowledge of tick species distribution in this region. Furthermore, the PCR detection of *T. ovis*, *T. luwenshuni*, and *A. ovis* DNA in *H. marginatum* provides fundamental molecular surveillance data for tick-borne pathogen monitoring in this region; however, because all examined ticks were engorged, the detected DNA may have originated from the residual host blood meal, and these findings do not demonstrate infection of the ticks or vector competence.

## Simple Summary

Ticks are important pathogen vectors. We collected 80 engorged *Hyalomma marginatum* ticks from goats in Guide County, Qinghai, China. Ticks were identified by morphology and *16S* rRNA/*cox1* sequencing. PCR detected *Theileria ovis* DNA in 5.0%, *Theileria luwenshuni* DNA in 17.5%, and *Anaplasma ovis* DNA in 6.3% of ticks. Because all ticks were engorged, the detected DNA may have come from residual goat blood rather than tick infection. These findings provide baseline surveillance data but do not prove vector competence. Studies using unfed ticks or salivary glands are needed.

## 1. Introduction

Ticks, as obligate hematophagous ectoparasitic arthropods, are important vectors of various zoonotic diseases worldwide. Ticks are vectors for a wide range of pathogens, including viruses, bacteria, and protozoa, causing numerous diseases in humans and animals such as tick-borne encephalitis, Lyme disease, and anaplasmosis [1,2,3]. As blood-feeding vectors, ticks pose fewer hazards only than mosquitoes and can sustain a wide range of pathogens of natural foci for a long time. Owing to their distribution worldwide, tick-borne diseases not only lead to massive economic losses to the livestock industry but also constitute severe threats to human and animal health [4]; therefore, ticks are considered one of the most significant vectors that threaten animal and human health. *Hyalomma marginatum* exhibits strong vectorial capacity and has been confirmed as a vector for numerous viruses, including Thogoto virus, Dhori virus, Crimean-Congo hemorrhagic fever virus (*Orthonairovirus haemorrhagiae*), West Nile virus (*Orthoflavivirus nilense*), African horse sickness virus (*Orbivirus alphaequi*), and Kyasanur Forest disease virus (*Orthoflavivirus kyasanurense*), in addition to the human spotted fever-causing *Rickettsia aeschlimannii* [5]. In recent years, novel viruses such as Volzhskoe tick virus, Balambala tick virus, Bole tick virus 4 (*Boletivirus hyalommae*), and *Hyalomma marginatum* rhabdovirus have been identified in *H. marginatum* [6], further expanding the potential risks that tick vectors pose to human and animal health.

Situated in the northeastern region of the Qinghai–Tibet Plateau, Qinghai Province is a significant hub for cattle production in China. Ticks and tick-borne illnesses can thrive and spread there thanks to its distinct biological setting and abundant biodiversity. Prior research on tick-borne illnesses and tick fauna in Qinghai Province primarily focused on common local taxa such as *Dermacentor* and *Haemaphysalis* [7]. However, with climate change, animal trade, and migration, the risk of invasion by new tick species is increasing. Recently, a research team collected and identified *H. marginatum* for the first time in Jianzha County, Qinghai Province, indicating that the geographical distribution of this important vector tick has extended to the northeastern margin of the Qinghai–Tibet Plateau [8]. Although the present findings confirm the presence of *H. marginatum* in Qinghai Province, a systematic investigation and assessment of whether DNA of important tick-borne pathogens such as *Theileria* and *Anaplasma* species can be detected in this tick species remain lacking. Previous studies documented the prevalence of *Theileria* and *Anaplasma* infections in sheep flocks in Qinghai Province [9,10]; however, the primary vector reported in this region is *Haemaphysalis qinghaiensis* [11]. Whether DNA of the above pathogens can be detected in *H. marginatum*, and whether this tick species carries genotypes not previously reported in this region, are questions worthy of further investigation and have reference value for local tick-borne disease surveillance. To address this, ticks parasitizing the perineal region of goats were collected and identified using morphological and molecular approaches. PCR assays were then employed to detect the nucleic acids of *Theileria* and *Anaplasma* in tick specimens collected from a farm in Hedong Township, Guide County, Qinghai Province (101.4521° E, 36.0472° N). The results provide baseline molecular surveillance data on tick-borne pathogens in this area and may inform the development of local tick-borne disease prevention and control strategies.

## 2. Materials and Methods

### 2.1. Tick Collection

**Figure 1:**
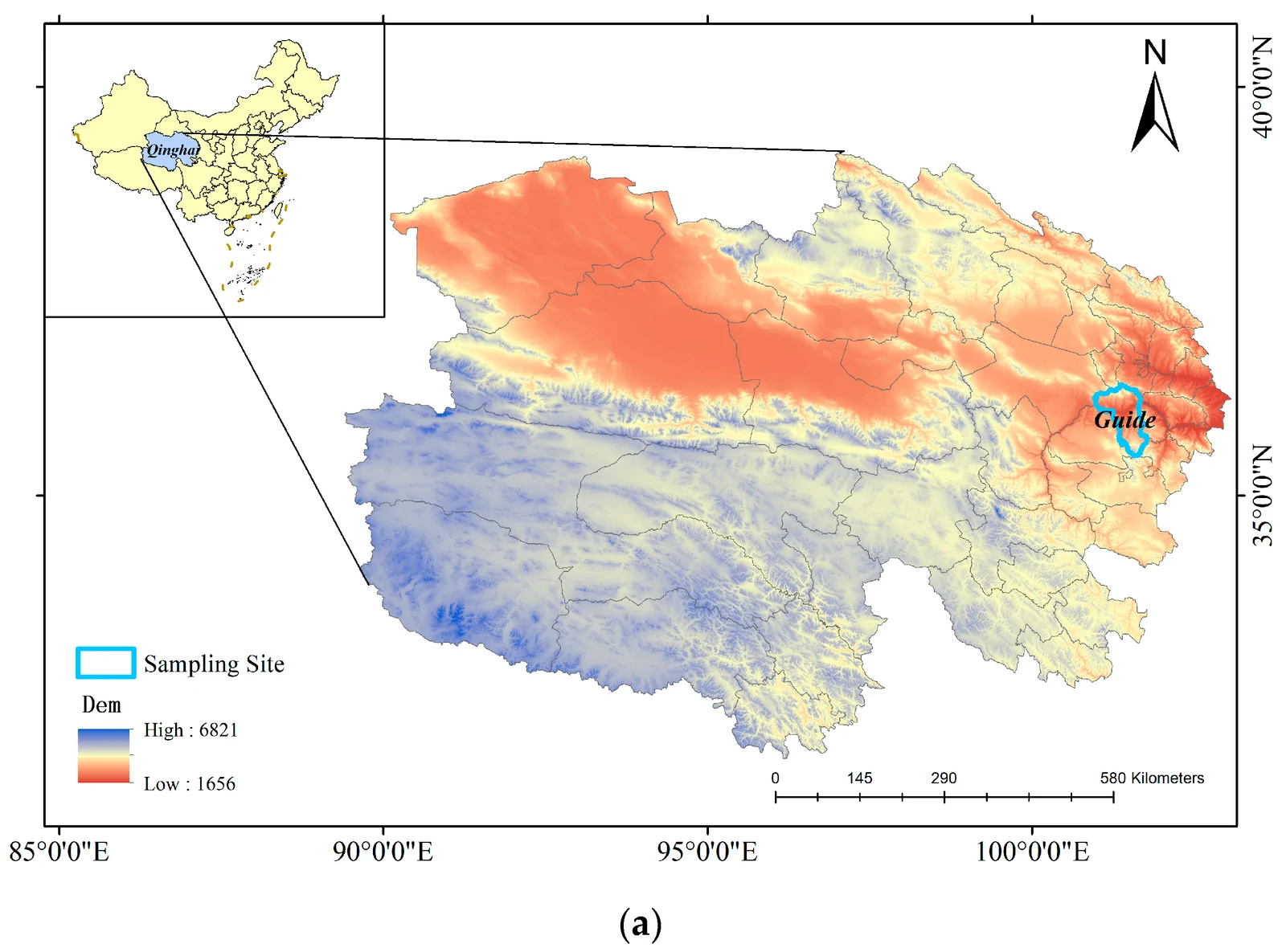

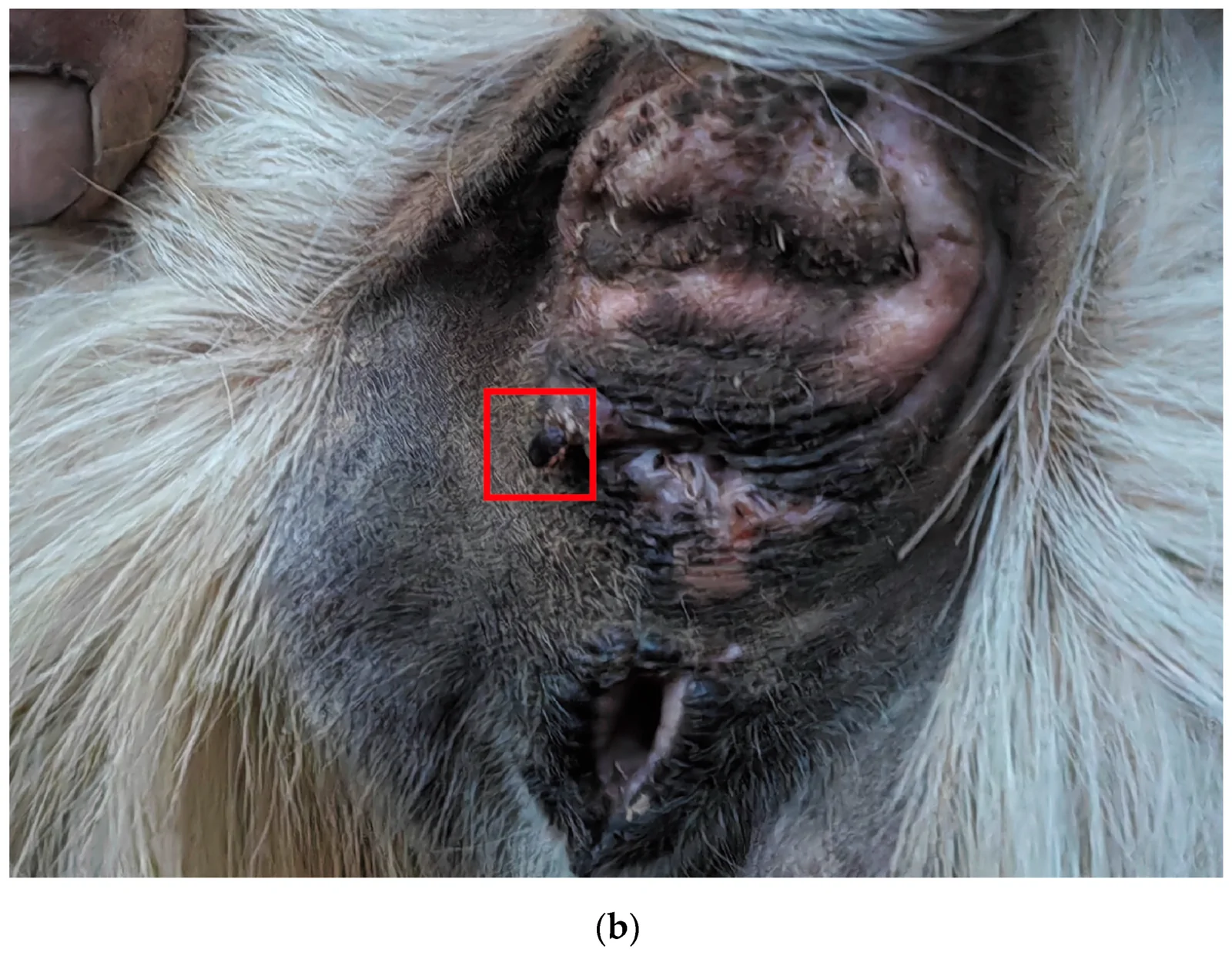
Sampling locations and collection sites for the goats in this study. (**a**) Distribution map of the sampling sites in Guide County, Qinghai Province, China. The blue box indicates the sampling area of this study. (**b**) Field photograph showing a parasitic tick found in the perineal region of a goat in Guide County, Qinghai Province. The red box indicates the attachment site of the tick on the host’s perineal region.

During April 2024, a total of 45 engorged ticks were collected from the perineal region of 25 goats, and during April 2025, a total of 35 engorged ticks were collected from the perineal region of 20 goats. The perineal region was selected as the sampling site because, during the investigation, ticks were found parasitizing the perineal region of goats on a farm in Guide County, Qinghai Province (Figure 1). Their gross morphology differed markedly from that of *Haemaphysalis* and *Dermacentor* ticks, and they moved rapidly; therefore, these ticks were collected for systematic identification. In total, 45 goats (approximately 15% of the total goats on the farm) were sampled from a farm in Hedong Township, Guide County, Qinghai Province, and 80 ticks were collected, with 1–5 ticks per goat. The goats sampled in April 2024 and April 2025 were different individuals from the same farm. All 80 ticks were adults, comprising 59 females and 21 males. Ticks were carefully removed from the skin using forceps. Using forceps, ticks were carefully detached from the skin, placed individually into 50 mL centrifuge tubes, and the collection data were recorded. All specimens were preserved in 75% ethanol for subsequent morphological and molecular identification. The precise sampling coordinates were 101.4521° E, 36.0472° N. Voucher specimens have been deposited at the Plateau Zoonoses Laboratory, College of Animal Science and Veterinary Medicine, Qinghai University. Each tick was processed individually for DNA extraction and detection.

### 2.2. Morphological Identification

Ticks were removed immersed in 75% ethanol, washed with PBS, and examined under a Nikon SMZ1000 research stereomicroscope (Nikon Corporation, Tokyo, Japan) coupled with a Nikon Ri1 imaging system (Nikon Corporation, Tokyo, Japan). Genus and species identification was carried out following published keys [12,13], primarily relying on the morphology of the basis capituli, palps, scutum, spiracular plate, anal groove, coxae, and internal spur.

### 2.3. Molecular Identification

#### 2.3.1. DNA Extraction

Tick specimens that had been morphologically identified were placed into cryovials containing a small amount of liquid nitrogen for immersion, to ensure complete inactivation and embrittlement. The specimens were then ground into a fine powder using a pestle, with liquid nitrogen being replenished intermittently throughout the process to maintain the samples in a low-temperature, brittle state until thorough pulverization was achieved. To prevent cross-contamination between samples, the disposable pestle was discarded after each grinding procedure, and the mortar was thoroughly cleaned and sterilized between specimens. The ground tick tissue was used for DNA extraction following the instructions provided for the Blood/Cell/Tissue Genomic DNA Extraction Kit (TIANGEN, Beijing, China, #DP304-03). Centrifugation steps during the extraction process were performed using a refrigerated high-speed benchtop centrifuge (LaBogene A/S, Allerød, Denmark, #SCANSPEED 1730 R). An aliquot of 70 μL of the extracted DNA was stored at −20 °C for subsequent use.

#### 2.3.2. PCR Amplification and Sequencing

Referring to previously published studies [14,15], the tick mitochondrial *16S* rRNA and *cox1* genes were selected as molecular targets for tick species identification. The universal primers were synthesized by Sangon Biotech (Shanghai) Co., Ltd., Shanghai, China, and detailed primer sequences and their corresponding references are shown in Table 1. All 80 genomic DNA samples extracted from tick specimens were subjected to PCR amplification using the two sets of primers separately. The total volume of each reaction system was fixed at 25 μL, containing 12.5 μL of TaKaRa Premix Taq™ (TaKaRa, Dalian, China), 8.5 μL of RNase-free water (TaKaRa, Dalian, China), 2 μL of extracted DNA template, and 1 μL of each 10 μM forward and reverse primer. A negative control (RNase-free water replacing the DNA template) was included in each PCR run to monitor contamination. For the *16S* rRNA gene, the temperature cycling setup began with a 1 min pre-denaturation treatment at 94 °C. After that, 35 reaction cycles were performed, with 10 s of denaturation at 98 °C, 30 s of annealing at 55 °C, and 45 s of extension at 68 °C. Following cyclic amplification, a 10 min terminal extension at 68 °C was introduced and added after cyclic amplification. For the *cox1* gene, the cycling conditions were identical except that the annealing temperature was set to 56 °C and the extension time to 1 min. For electrophoretic detection, 8 μL amplified product was loaded into a 1.2% agarose gel, followed by constant-voltage electrophoresis at 120 V with a running duration of 40 min. The amplified fragments were visualized using a G:BOX gel imaging system (Syngene, Cambridge, UK). Positive amplicons were sent to Sangon Biotech (Shanghai) Co., Ltd., Shanghai, China, for sequencing. The obtained sequences were subjected to a BLAST (version 2.17.0+) similarity search in NCBI to confirm whether the sequences were the target gene and to identify the tick species.

### 2.4. PCR Detection and Sequencing of Theileria and Anaplasma

Based on references [16,17,18,19], a total of four PCR assays were employed, as described below: a genus-level nested PCR for *Theileria* spp. targeting the *18S* rRNA gene, comprising a first-round primer pair (Piro1-S/Piro3-AS) and a second-round primer pair (PIRO-A/PIRO-B); a species-specific PCR for *T. luwenshuni* targeting the *18S* rRNA gene (primer pair P3/P4); a species-specific PCR for *T. uilenbergi* targeting the *18S* rRNA gene (primer pair YF/YR); and (iv) a genus-level nested PCR for *Anaplasma* spp. targeting the *groEL* gene, comprising a first-round primer pair (gro607F/gro1249R) and a second-round primer pair (gro677F/gro1121R). All primer pairs, including the first- and second-round pairs of the two nested assays, are listed in Table 1 together with their target genes, assay levels, amplicon sizes and the corresponding references. All primers were synthesized by Sangon Biotech (Shanghai) Co., Ltd., Shanghai, China,.

Each of the four PCR assay systems described above was applied to all 80 purified DNA isolates. For the two genus-level nested PCR systems (universal *Theileria* and universal *Anaplasma*), amplification was performed in two successive rounds, and the first-round amplicon was used as the template for the second-round reaction; for the two species-specific PCRs (*T. luwenshuni* and *T. uilenbergi*), a single-round amplification was performed. The total reaction volume and thermal cycling program for each pathogen were consistent with the common parameters used for tick *16S* rRNA identification described above, with differences only in the annealing temperature and extension time, which are specified for each primer pair and each round in Table 1. A negative control (RNase-free water replacing the DNA template) and a positive control (known DNA positive for the target genes) were included in each PCR run of each assay to monitor reaction efficiency and cross-contamination. To prevent contamination, template addition, amplification, and product detection were performed in separate areas, and filter-tipped pipette tips were used throughout. After amplification, an aliquot of the PCR products was subjected to electrophoresis on a 1.2% agarose gel. Band patterns were visualized using a gel imaging system. All positive amplicons were sent to Sangon Biotech (Shanghai) Co., Ltd., Shanghai, China, for sequencing.

All the obtained sequences were subjected to a BLAST (version 2.17.0+) similarity search in NCBI to confirm whether they were the target genes and to identify the *Anaplasma* and *Theileria* species.

### 2.5. Phylogenetic Analysis

The obtained nucleotide sequences were submitted to the NCBI-BLAST server for homology comparison with reference sequences deposited in GenBank, whereas multiple sequence alignments were performed in MEGA 11.0.13 with default parameters. For each dataset, the best-fit nucleotide substitution models were determined using the Find Best DNA/Protein Models (ML) function in MEGA 11.0.13 based on the Bayesian Information Criterion (BIC). Accordingly, T92+G, TN93+G, K2, and T92 were selected for the tick *16S* rRNA, tick *cox1*, *Theileria 18S* rRNA, and *Anaplasma groEL* datasets, respectively. Final alignment lengths were 381 bp (tick *16S* rRNA), 645 bp (tick *cox1*), 299 bp (*Theileria 18S* rRNA), and 334 bp (*Anaplasma groEL*); positions containing gaps or missing data were treated using the partial deletion option with a 90% site coverage cutoff. Phylogenetic trees of the tick *16S* rRNA, tick *cox1*, *Theileria 18S* rRNA, and *Anaplasma groEL* genes were then constructed separately using the Maximum Likelihood method with 1000 bootstrap replicates to assess tree topology reliability. Reference sequences were retrieved from GenBank based on highest sequence similarity and geographic representativeness.

### 2.6. Statistical Analysis

Molecular detection results of pathogens in tick samples were statistically analyzed using R software (version 4.6.1). The DNA detection rate of each pathogen was calculated together with its exact (Clopper–Pearson) 95% binomial confidence interval, using the binom package (version 1.1-2; function binom.confint). The co-occurrence association between pairs of pathogens within individual ticks was assessed using the Centered Jaccard index, defined as J − E(J), where J = a/(a + b + c) is the Jaccard index for each pathogen pair (a = number of ticks positive for both pathogens; b and c = numbers positive for only one of the two), and E(J) is the Jaccard index expected under the null hypothesis of independence, calculated from the marginal detection rates of the two pathogens as E(J) = p_1_p_2_/(p_1_ + p_2_ − p_1_p_2_); positive values thus indicate co-detection more frequent than expected by chance, and negative values less frequent than expected [20]. Ninety-five percent confidence intervals for the Centered Jaccard index were obtained by nonparametric bootstrap resampling with 100,000 replicates (boot package, version 1.3-32; functions boot and boot.ci, percentile method), with the individual tick sample as the resampling unit. Because multiple ticks originated from the same goat, tick-level resampling may not fully account for host clustering, which is acknowledged as a limitation of this analysis. Additionally, Fisher’s exact test (two-sided; stats package, function fisher.test) was employed to determine whether the co-detection between any two pathogens was statistically significant.

## 3. Results

### 3.1. Morphological Identification Results

**Figure 2:**
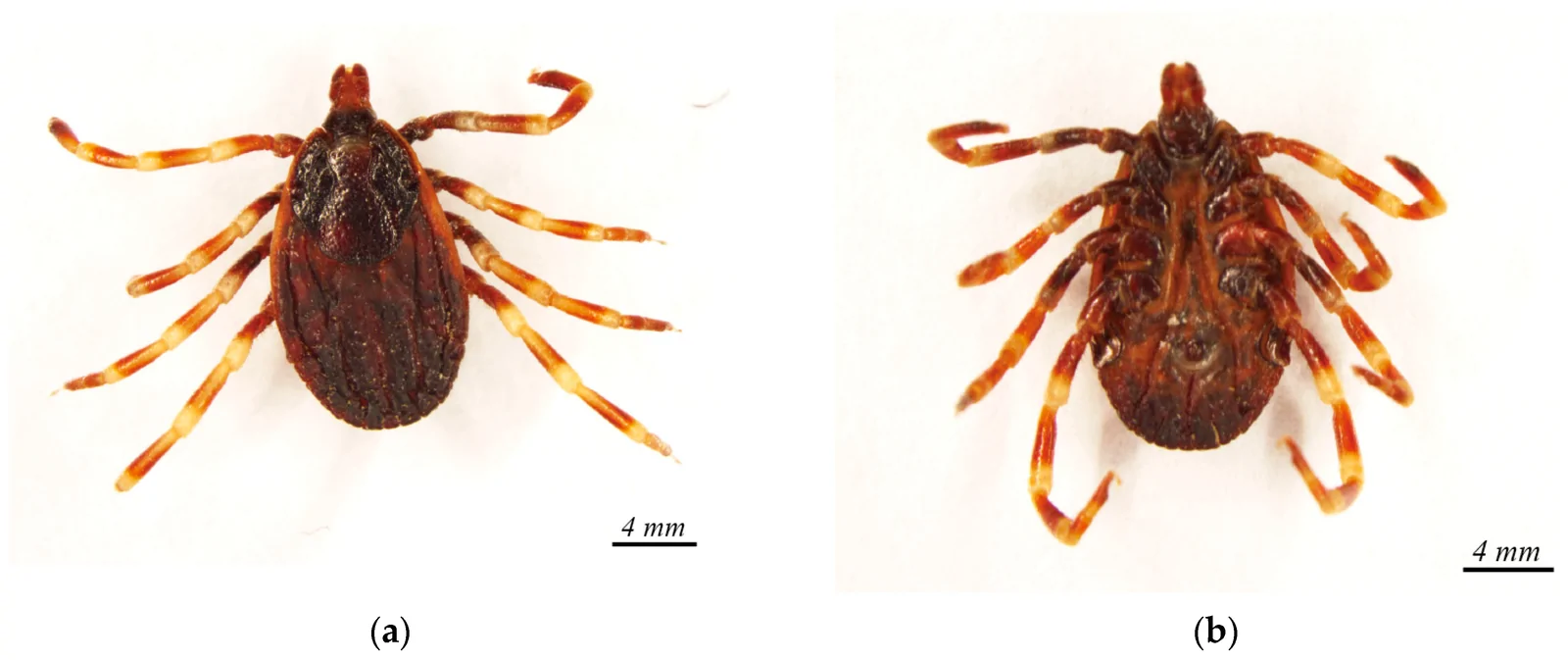

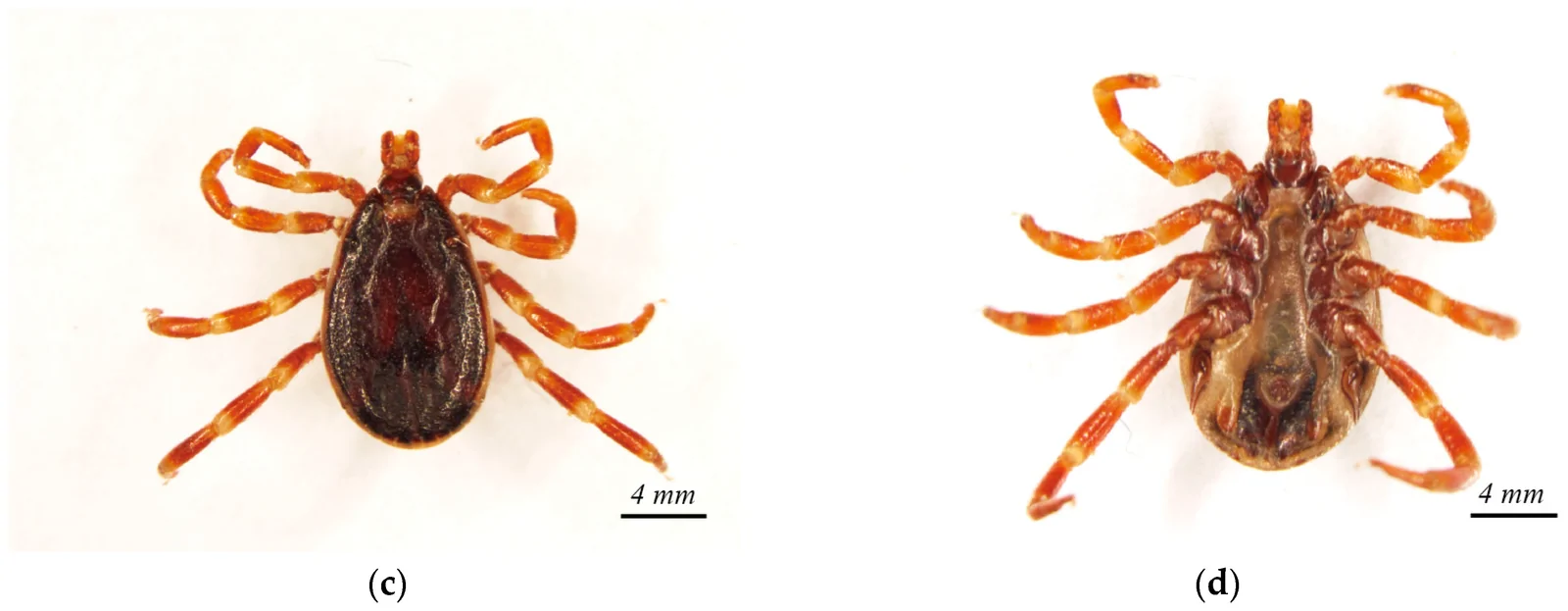
Morphological characteristics of *H. marginatum*. (**a**) Dorsal view of a female *H. marginatum*. (**b**) Ventral view of a female *H. marginatum*. (**c**) Dorsal view of a male *H. marginatum*. (**d**) Ventral view of a male *H. marginatum*.

Microscopic observation revealed that the female ticks exhibited the following morphological characteristics: basis capituli triangular; cervical grooves distinct and relatively deep, but not reaching the posterior margin of the scutum; palps narrow and elongated; scutum dark-coloured with a distinctly curved posterior margin, bearing few unevenly distributed punctations; spiracular plate comma-shaped; festoons present; and each leg segment with pale bands on the dorsal side. The features of the male ticks were as follows: basis capituli triangular; cervical grooves distinct and deep; palps narrow and elongated; scutum dark-coloured, covering the entire dorsal surface of the body, with few unevenly distributed punctations; festoons present; posterior median groove present but shallow; and each leg segment with pale annular bands on the dorsal side. These characteristics were consistent with the morphological features of *H. marginatum*. The detailed morphological features are shown in Figure 2.

### 3.2. Molecular Identification Results

**Figure 3:**
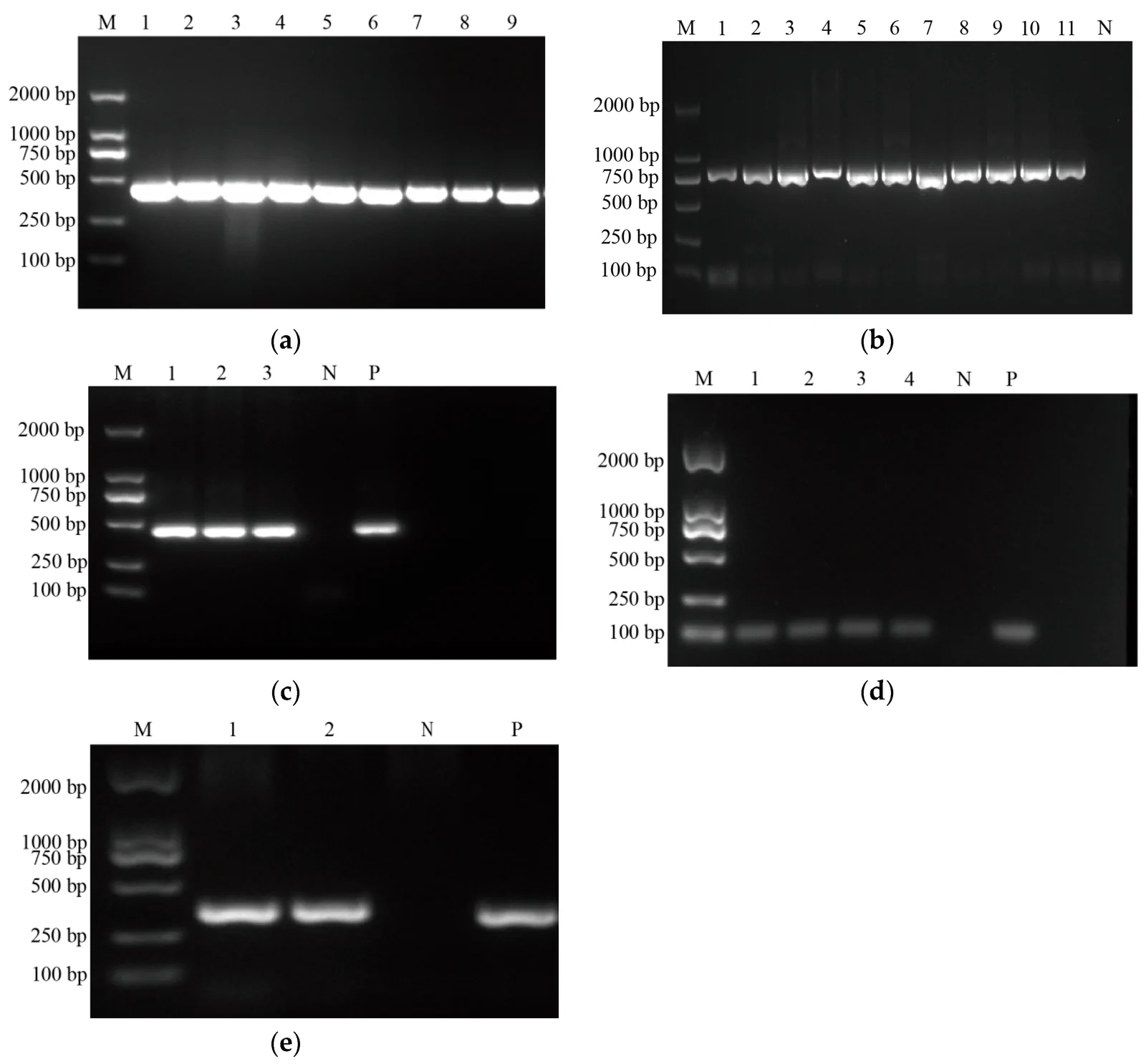
Electrophoresis results of PCR products for tick identification, *Theileria*, and *Anaplasma*. (**a**) Electrophoresis of *16S* rRNA gene PCR amplification products from partial tick samples collected in Guide County. (**b**) Electrophoresis of *cox1* gene PCR amplification products from partial tick samples collected in Guide County. (**c**) Electrophoresis of second-round amplification products from partial samples using *Theileria* primers. (**d**) Electrophoresis of amplification products of *T. luwenshuni* from partial samples. (**e**) Electrophoresis of second-round amplification products from partial samples using universal *Anaplasma* primers. (M: DL2000 DNA marker; 1–11: sample numbers; N: negative control; P: positive control).

Amplification of the tick *16S* rRNA gene yielded products that, upon electrophoresis on a 1.2% agarose gel, displayed target bands at approximately 450 bp, consistent with the expected fragment size (partial electrophoretograms are shown in Figure 3a). Amplification of the tick *cox1* gene produced target bands at approximately 709 bp, matching the expected fragment size (partial electrophoretograms are shown in Figure 3b). All positive amplicons from the 80 tick samples were successfully sequenced for both genes. Sequence analysis of the obtained *16S* rRNA gene fragments revealed that all 80 sequences exhibited 98.1–99.7% similarity to the corresponding reference sequences of *H. marginatum* available in GenBank; similarly, the *cox1* gene sequences showed 97.7–99.7% similarity to reference sequences of *H. marginatum*. Based on both morphological and molecular identification, all tick specimens were confirmed as *H. marginatum*. Representative sequences for each gene were selected and deposited in GenBank under accession numbers PZ675184-PZ675188 (*16S* rRNA) and PZ902087-PZ902089 (*cox1*).

### 3.3. PCR Detection Results for Theileria and Anaplasma

All 80 tick tissue samples were subjected to PCR amplification using different primer sets. Nested PCR was carried out to amplify the *Theileria 18S* rRNA gene, and the secondary amplicons were resolved via 1.2% agarose gel electrophoresis, producing expected bands of 430 bp. *T. ovis* DNA was detected in four tick samples, with a DNA detection rate of 5.0% (4/80) among the tick samples. Sequencing and BLAST analysis revealed 99.2–100% similarity to the reference sequences of *T. ovis*, confirming the presence of *T. ovis* DNA in the tick samples (partial electrophoretograms are shown in Figure 3c). PCR amplification using *T. luwenshuni* primers produced target bands at approximately 103 bp. *T. luwenshuni* DNA was detected in 14 tick samples, with a DNA detection rate of 17.5% (14/80). Sequence alignment showed 96.4–100% similarity to *T. luwenshuni* reference sequences, indicating the presence of *T. luwenshuni* DNA in these tick specimens (partial electrophoretograms are shown in Figure 3d). Species assignment was based on the highest BLAST similarity (query coverage 96%) using the 90 bp sequence retained after quality trimming. Given the very short amplicon length (~103 bp), the discriminatory power of this assay is limited, and species-level assignment and any phylogenetic inference based on this fragment should be interpreted with caution. In contrast, no target bands were observed in any samples following amplification with *T. uilenbergi* primers, indicating that *T. uilenbergi* DNA was not detected in the collected tick specimens. All positive amplicons were sequenced, and representative sequences for each gene were deposited in GenBank under accession numbers PZ683483–PZ683485 (*T. ovis 18S* rRNA) and QB078840 and PZ675192 (*T. luwenshuni 18S* rRNA).

Using universal *Anaplasma* primers, nested PCR was performed on the 80 tick tissue samples. The secondary PCR amplicons were subjected to 1.2% agarose gel electrophoresis analysis, and a specific target band of approximately 364 bp was observed, matching the expected fragment size (partial electrophoretograms are shown in Figure 3e). *A. ovis* DNA was detected in five tick samples, with a DNA detection rate of 6.3% (5/80) among the tick samples. Sequencing of the PCR products followed by BLAST analysis revealed 99.2–100% similarity to the reference sequences of *A. ovis*, confirming the presence of *A. ovis* DNA in the tick samples. All positive amplicons were sequenced, and representative sequences were deposited in GenBank under accession numbers PZ706621–PZ706622 (*A. ovis groEL*).

**Figure 4:**
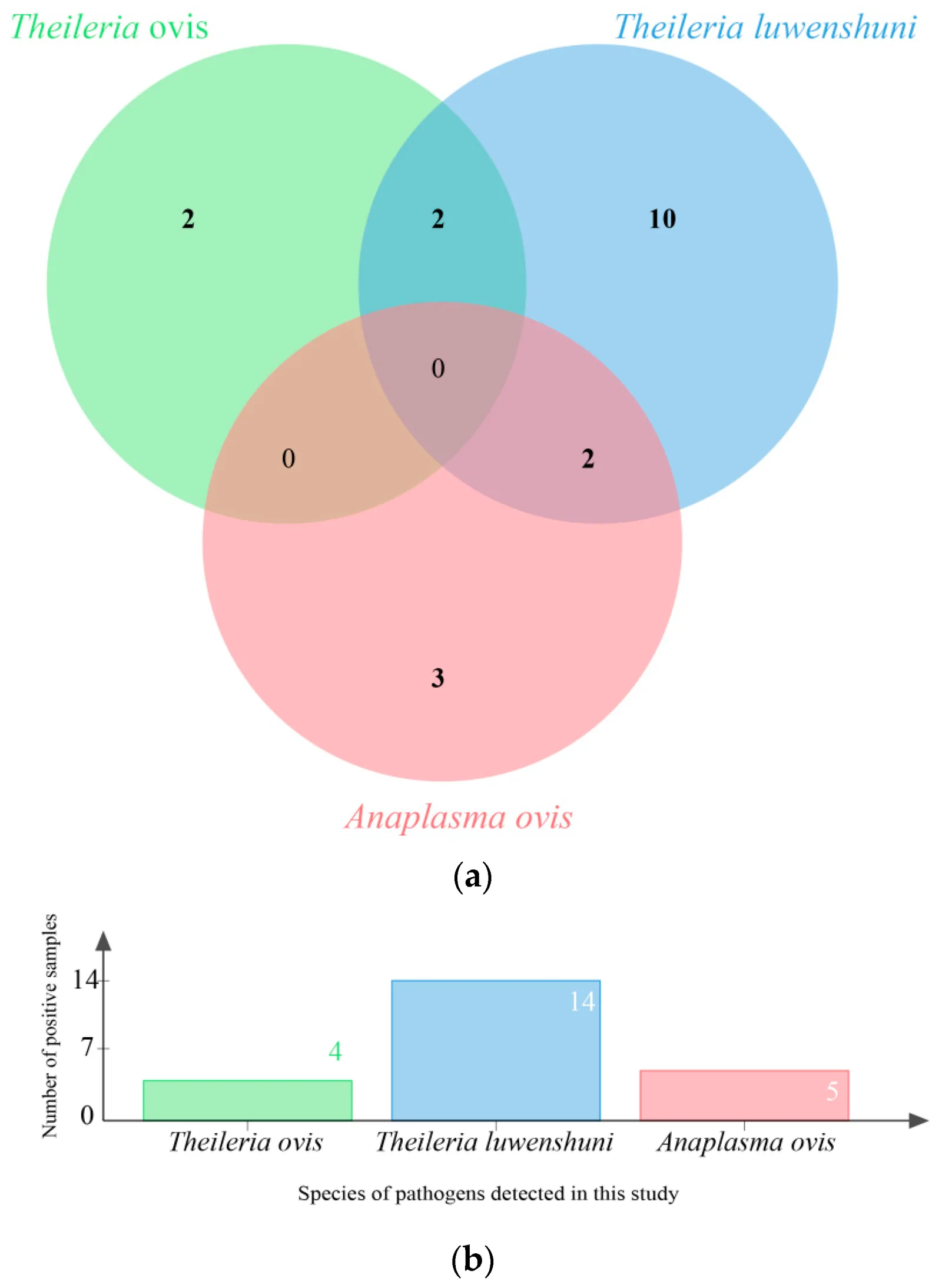
Detection of three pathogen nucleic acids in *H. marginatum* tick samples. (**a**) Venn diagram illustrating the distribution of nucleic-acid-positive tick samples among the 80 specimens. The green, blue, and pink circles correspond to *T. ovis*, *T. luwenshuni*, and *A. ovis*, respectively. Non-overlapping sections indicate the number of samples positive for a single pathogen, whereas overlapping sections represent samples with co-detection of two pathogens. No samples were positive for all three pathogens simultaneously, nor were any samples positive for both *T. ovis* and *A. ovis.* (**b**) Bar graph showing the total number of tick samples testing nucleic-acid-positive for each pathogen.

Among the 80 *H. marginatum* tick samples tested, nucleic acids of three pathogens—*T. ovis*, *T. luwenshuni*, and *A. ovis*—were detected. Specifically, *T. luwenshuni* nucleic acid was detected in 14 tick samples (DNA detection rate of 17.5%), of which 10 were positive exclusively for this pathogen; *T. ovis* nucleic acid was detected in 4 samples (DNA detection rate of 5.0%), of which 2 were positive exclusively for this pathogen; and *A. ovis* nucleic acid was detected in 5 samples (DNA detection rate of 6.3%), of which 3 were positive exclusively for this pathogen. Regarding co-detection, 2 samples tested positive for both *T. ovis* and *T. luwenshuni*, and 2 samples tested positive for both *T. luwenshuni* and *A. ovis*. No samples tested positive for both *T. ovis* and *A. ovis*, nor were any samples found positive for all three pathogens simultaneously (Figure 4). Since all examined ticks were engorged, the detection and co-detection of pathogen nucleic acids may have originated from the residual blood meal of the host rather than indicating infection or carriage within the ticks themselves.

### 3.4. Phylogenetic Analysis Results

**Figure 5:**
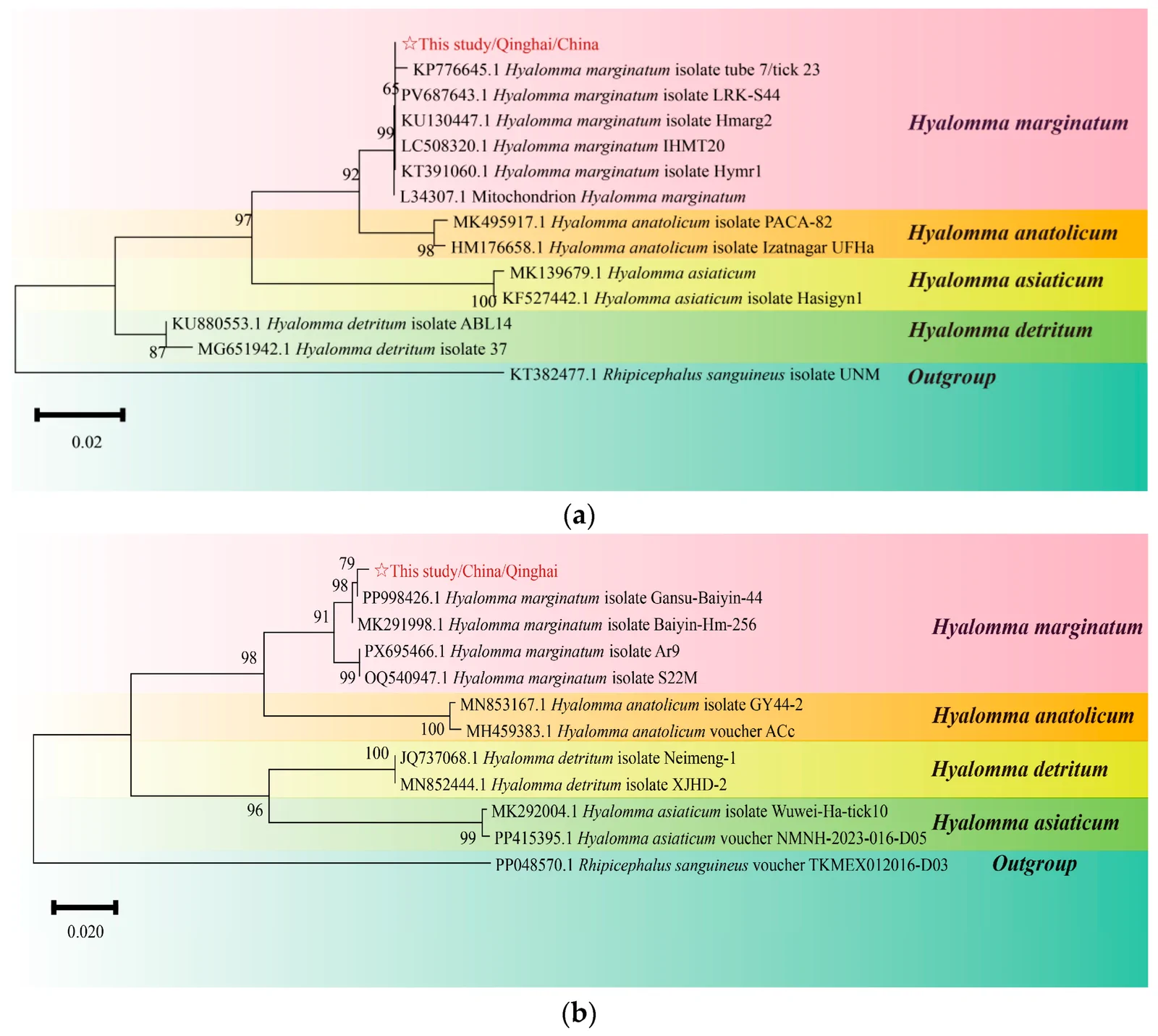

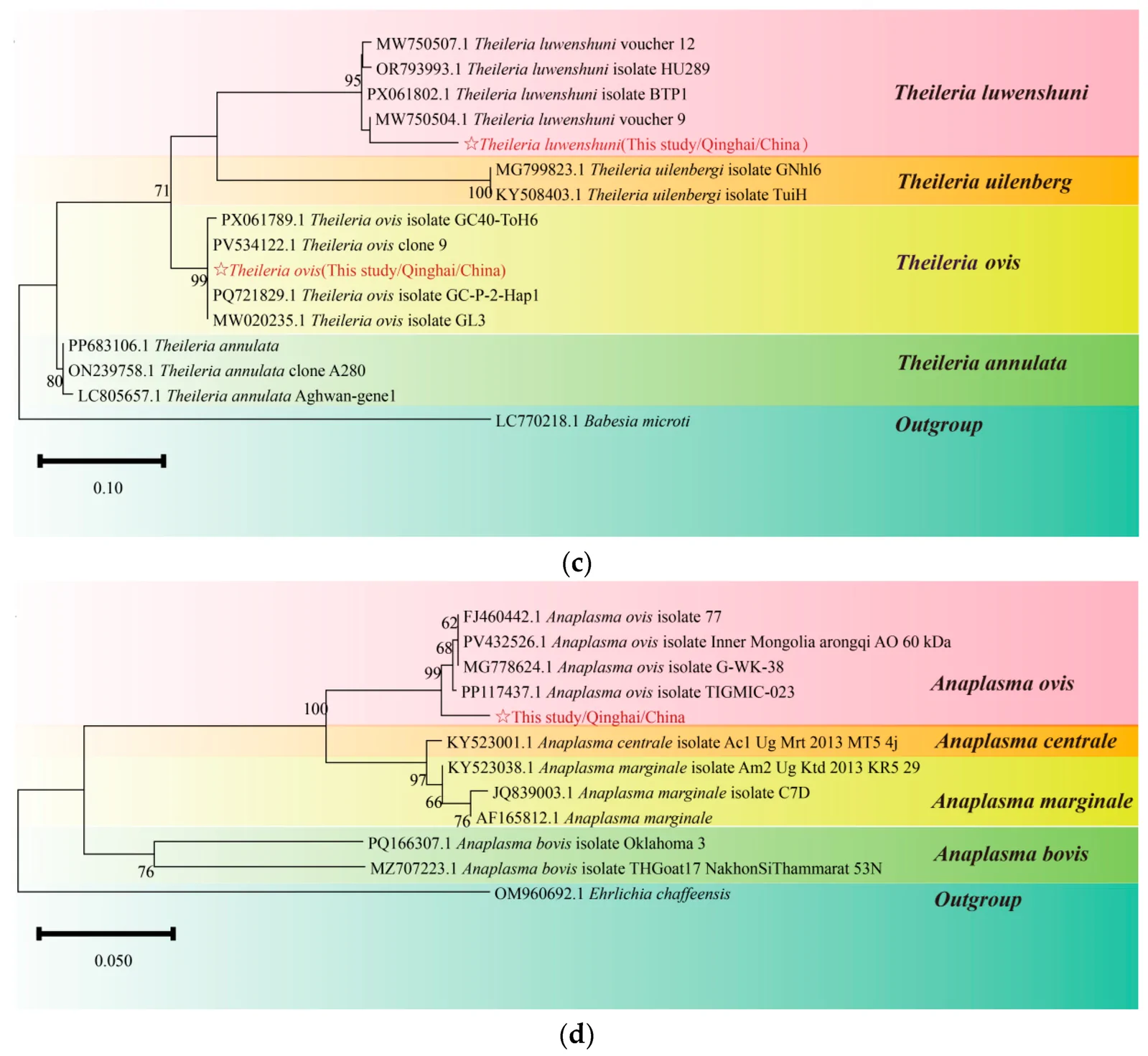
Phylogenetic trees were constructed using the Maximum Likelihood method in MEGA 11. Bootstrap support values are shown at each node, and the scale bar indicates genetic distance. The five-pointed star (☆) denotes the sequences generated in the present study from Qinghai, China. (**a**) Phylogenetic tree based on the *16S* rRNA gene of *H. marginatum*, with *Rhipicephalus sanguineus* (KT382477.1) defined as the outgroup (teal). Branches are colour-coded according to species: *H. marginatum* (pink), *H. anatolicum* (orange), *H. asiaticum* (light-yellow), and *H. detritum* (green). (**b**) Phylogenetic tree based on the *cox1* gene of *H. marginatum*, with *Rhipicephalus sanguineus* (PP048570.1) as the outgroup (teal). Branches are colour-coded according to species: *H. marginatum* (pink), *H. anatolicum* (orange), *H. detritum* (light-yellow), and *H. asiaticum* (green). (**c**) Phylogenetic tree of *Theileria* spp., with *Babesia microti* (LC770218.1) serving as the outgroup (teal). Branches are colour-coded according to species: *T. luwenshuni* (pink), *T. uilenbergi* (orange), *T. ovis* (yellow), and *T. annulata* (green). (**d**) Phylogenetic tree of *Anaplasma* spp., with *Ehrlichia chaffeensis* (OM960692.1) as the outgroup (teal). Branches are colour-coded according to species: *A. ovis* (pink), *A. centrale* (orange), *A. marginale* (yellow), and *A. bovis* (green).

Phylogenetic analysis based on representative sequences revealed that (Figure 5) the *H. marginatum 16S* rRNA gene sequences obtained in this study from Qinghai clustered with multiple published reference sequences of *H. marginatum* (KP776645, PV687643, KU130447, etc.) and fell within the same clade as, and showed high sequence similarity to, the strain from France (KP776645). The *H. marginatum cox1* gene sequences clustered with multiple published reference sequences of *H. marginatum* (PP998426, MK291998, PX695466, etc.) and were placed in the same clade as, with the highest sequence similarity to, the strain from Gansu, China (PP998426). The *T. ovis 18S* rRNA gene sequences detected in tick samples clustered with multiple published reference sequences of *T. ovis* (PX061789, PQ721829, MW020235, PV534122) and were placed in the same clade as, with high sequence similarity to, the strain from Qinghai, China (PQ721829). The *T. luwenshuni 18S* rRNA gene sequences detected in tick samples clustered with multiple published reference sequences of *T. luwenshuni* (MW750507, OR793993, PV283097, MW750504) and were placed in the same clade as, with high sequence similarity to, the strain from Hunan, China (MW750504). The *A. ovis groEL* gene sequences detected in tick samples clustered with multiple published reference sequences of *A. ovis* (FJ460442, PV432526, MG778624, PP117437) and were placed in the same clade as, with the highest sequence similarity to, the strain from Beijing, China (PP117437). It should be noted that, because partial and, in some cases, relatively short gene fragments were analyzed, the phylogenetic placements described above reflect sequence similarity and topology within the reconstructed trees and do not necessarily imply direct epidemiological or geographical relationships. The representative sequences used for the above analyses have been deposited in GenBank under accession numbers PZ675184 (*16S* rRNA), PZ902087 (*cox1*), PZ683483 (*T. ovis 18S* rRNA), PZ675192 (*T. luwenshuni 18S* rRNA), and PZ706621 (*A. ovis groEL*).

### 3.5. Statistical Analysis Results

**Figure 6:**
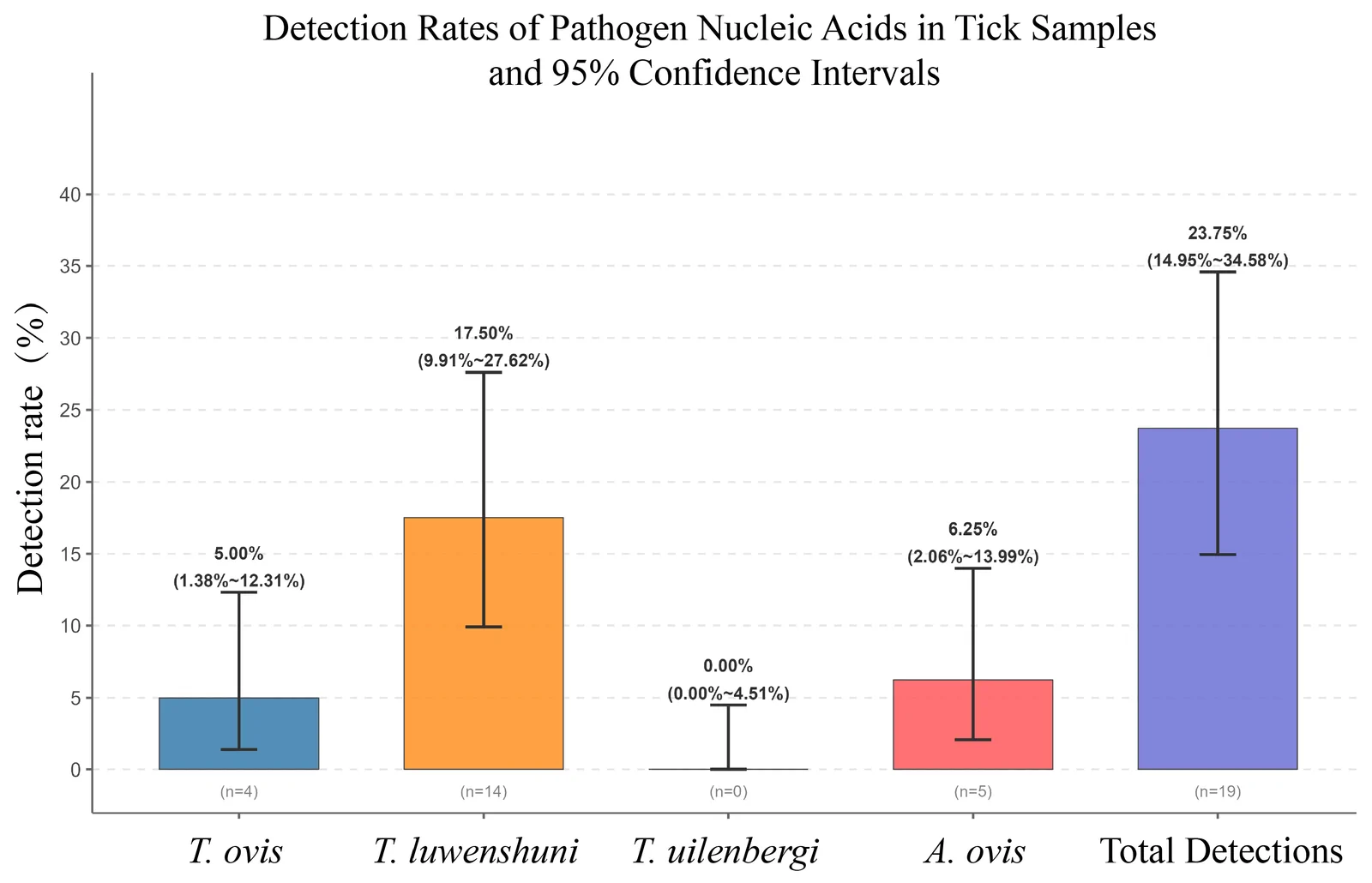
Nucleic acid detection rates and Clopper–Pearson 95% exact confidence intervals for four pathogens among the 80 tick specimens identified as *H. marginatum*. Note: The ordinate represents the pathogen nucleic acid detection rate (%) among tick samples; error bars indicate the 95% exact confidence intervals; *n* denotes the number of samples positive for the corresponding pathogen nucleic acid; overall nucleic acid positivity rate refers to the proportion of tick specimens in which at least one target pathogen nucleic acid was detected.

All 80 tick specimens were subjected to PCR-based nucleic acid detection for four pathogens. The 95% confidence intervals of the detection rates were calculated using the exact (Clopper–Pearson) binomial method (Figure 6). The nucleic acid detection rate for *T. ovis* among tick samples was 5.0% (4/80, 95% CI: 1.38–12.31%); *T. luwenshuni* exhibited the highest detection rate at 17.5% (14/80, 95% CI: 9.91–27.62%); the detection rate for *A. ovis* was 6.3% (5/80, 95% CI: 2.06–13.99%); and *T. uilenbergi* was not detected, with a one-sided 97.5% upper confidence limit of 4.51%. A total of 19 tick specimens tested positive for at least one pathogen, yielding an overall nucleic acid positivity rate of 23.8% (19/80, 95% CI: 14.95–34.58%) among tick samples. The relatively wide confidence intervals for each pathogen suggest appreciable sampling random error, likely attributable to the limited sample size. Co-detection associations between pathogen pairs were assessed using the Centered Jaccard index, which quantifies the deviation of the observed Jaccard similarity from the value expected under the null hypothesis of independence (a positive value indicates co-detection more frequently than expected by chance), with 95% confidence intervals obtained by nonparametric bootstrap resampling (100,000 replicates, with the individual tick specimen as the resampling unit) (Table 2). All *p* values were derived from two-sided Fisher’s exact tests. The Centered Jaccard index for *T. ovis* and *T. luwenshuni* was 0.085 (95% CI: −0.032 to 0.263, *p* = 0.139), and that for *A. ovis* and *T. luwenshuni* was 0.069 (95% CI: −0.042 to 0.238, *p* = 0.209). No tick specimen was simultaneously positive for *T. ovis* and *A. ovis* (Centered Jaccard index = −0.029, *p* = 1.000); this pair was therefore not included in the co-detection analysis. *T. uilenbergi* was likewise excluded from all pairwise analyses because it was not detected. For both pairwise comparisons, the bootstrap 95% confidence intervals included zero. These results are presented as descriptive measures of co-detection patterns in this tick sample set; formal inferential interpretation is limited by the clustered sampling design (multiple ticks collected from the same host), which the tick-level resampling does not fully account for, as well as by the fully engorged status of all ticks.

## 4. Discussion

During an epidemiological survey of blood-borne protozoan diseases in goats in Guide County, Qinghai Province, ticks were observed parasitizing the perineal region of the animals. These ticks exhibited morphological characteristics distinctly different from those of *Haemaphysalis* and *Dermacentor* species, and displayed rapid locomotion, prompting their collection for systematic identification. Through morphological and molecular analyses, the ticks were identified as *H. marginatum*. Furthermore, nucleic acids of *T. ovis*, *T. luwenshuni*, and *A. ovis* were detected in these tick samples. These results document the detection of these pathogens in engorged *H. marginatum* tick samples from Guide County, Qinghai Province, thereby enriching the molecular surveillance data of tick species and tick-borne pathogens in this region. However, it should be noted that all tick specimens were collected from a single farm in Hedong Township, Guide County; therefore, the findings are representative only of this localized area. Nevertheless, this study provides a preliminary scientific basis for the monitoring and control of tick-borne diseases in the local area.

*H. marginatum* is a tick species of significant medical and veterinary importance, widely distributed across Eurasia and northern Africa. In recent years, its geographical range has shown a marked expansion trend, driven by factors such as climate change, host migration, and international trade [21]. The discovery of this tick species in Guide County, Qinghai Province, in the present study corroborates a previous report of *H. marginatum* on the body surface of goats in Jianzha County, Qinghai Province [8], indicating the presence of *H. marginatum* on the northeastern margin of the Qinghai–Tibet Plateau. Guide County is situated in the Yellow River valley, characterized by a relatively mild climate and high vegetation coverage, which may provide suitable microhabitats for *H. marginatum*. Further dispersal of this tick species may be driven by factors such as migratory bird movements and cross-regional livestock transportation [22]; therefore, long-term monitoring of its population dynamics and distribution boundaries should be reinforced in the future. Notably, the phylogenetic analyses in the present study revealed an apparent discrepancy between the two mitochondrial markers examined. The *H. marginatum 16S* rRNA sequences obtained in this study showed high sequence similarity to a strain from France (KP776645), whereas the *cox1* sequences—a marker generally considered more discriminative at the intraspecific level in arthropods—clustered with a strain from the neighbouring province of Gansu, China (PP998426). This incongruence most likely reflects the limited resolving power of these short, partial gene fragments, in which topological relationships can be affected by incomplete lineage sorting, historical introgression, and the sparse geographic coverage of available reference sequences, rather than a genuinely dual European and local origin of the tick population. Nevertheless, given that *H. marginatum* is widely distributed across the Palearctic region and its dispersal has been repeatedly linked to livestock movement and migratory birds, the introduction of genetically divergent lineages into Qinghai through livestock importation or inter-provincial animal trade cannot be excluded. Further studies based on larger sample sizes and more variable markers (e.g., complete mitochondrial genomes) are needed to clarify the phylogeographic history of *H. marginatum* in western China. *Theileria* and *Anaplasma* are typical tick-borne pathogens that cannot be transmitted horizontally between hosts under natural conditions without vector involvement. The transmission cycle relies on the vector tick to facilitate cross-host transmission: ticks acquire pathogens by feeding on infected hosts, where the pathogens undergo development and proliferation within the tick; subsequently, when the tick feeds on a susceptible host, the pathogens are transmitted via the tick’s saliva, thereby completing the cycle and enabling circulation among host populations.

In this study, the DNA detection rate of *T. ovis* in *H. marginatum* tick samples was 5.0% (4/80), while that of *T. luwenshuni* was 17.5% (14/80), with a DNA co-detection rate of 2.5% (2/80) for *T. ovis* and *T. luwenshuni*. These findings indicate that DNA of these two *Theileria* species can be detected in *H. marginatum*. However, *T. uilenbergi*, a species commonly reported in local sheep populations, was not detected in this sample set; given the limited sample size, low-level occurrence cannot be excluded. *T. ovis* and *T. luwenshuni* are major pathogens responsible for theileriosis in sheep, with clinical manifestations including high fever, anemia, emaciation, generalized lymphadenopathy, as well as gastrointestinal and respiratory symptoms. Without timely treatment, the disease can increase mortality in sheep, leading to substantial economic losses to the sheep industry [23,24]. Recent research conducted in Iran has revealed that *T. ovis* DNA can be detected in *H. marginatum* [25], which is consistent with the findings of the present study. In addition, the species-specific assay used here for *T. luwenshuni* amplifies a very short fragment (approximately 103 bp), which provides limited discriminatory information; therefore, the phylogenetic placement of these sequences should be interpreted with caution. Taken together, these results indicate that DNA of *T. ovis* and *T. luwenshuni* can be detected in engorged *H. marginatum* in this region; whether this reflects a genuine association between this tick species and these pathogens, rather than DNA derived from the residual host blood meal, requires further investigation.

The PCR detection rate of *A. ovis* in *H. marginatum* ticks was 6.3% (5/80), and the co-detection rate of *A. ovis* with *T. luwenshuni* was 2.5% (2/80). These results indicate that *A. ovis* DNA can be detected in this tick species. However, no other *Anaplasma* species were detected in this sample set, and low-level occurrence of these species cannot be excluded, given the limited sample size. *A. ovis* is the causative agent of ovine anaplasmosis, which can cause anemia, jaundice, abortion, and even death in severe cases, posing a serious threat to small ruminant farming [26]. In Qinghai Province, previous studies have reported *Anaplasma* in ticks [27], but detection of *A. ovis* in *H. marginatum* has not been documented. Our finding is consistent with a recent molecular survey in Pakistan, where *A. ovis* DNA was detected in *H. marginatum* [28], further supporting that *A. ovis* nucleic acid can be detected in this tick species. Taken together with the results above, *A. ovis* DNA can be detected in engorged *H. marginatum* in this region; however, whether this reflects a genuine association between this tick species and *A. ovis*, rather than DNA derived from the residual host blood meal, requires further investigation.

The findings of this study are limited to the detection of pathogen DNA in tick samples and do not confirm the vector competence of *H. marginatum*. Nevertheless, *H. marginatum* is a recognized vector of several pathogens of medical and veterinary importance; vector competence, however, is pathogen-specific and cannot be inferred from the molecular detection of pathogen DNA in ticks. In other regions, *H. marginatum* is a confirmed vector of viruses such as Crimean-Congo hemorrhagic fever virus (CCHFV) and can harbor zoonotic bacteria such as *Rickettsia aeschlimannii*. CCHFV is a highly pathogenic hemorrhagic fever virus with case–fatality rates of up to 10–40% [29], and cases have been reported in Inner Mongolia, China [30]. Although these pathogens were not tested for in the present study, the emergence of *H. marginatum* in Qinghai Province and its documented role in the transmission of several pathogens support enhanced surveillance for tick-borne pathogens in local human populations and livestock, particularly early warning for highly pathogenic agents such as CCHFV.

In summary, this study represents the first molecular detection of *T. ovis*, *T. luwenshuni*, and *A. ovis* DNA in *H. marginatum* from Guide County, Qinghai Province, providing preliminary molecular surveillance data for this region. However, it should be noted that all tick specimens were collected from a single farm in Hedong Township, Guide County, with a limited sample size (80 ticks from 45 goats); therefore, the findings are representative only of this localized area and cannot be extrapolated to the entire Qinghai Province. In addition, ticks collected in 2024 and 2025 were pooled for analysis, and multiple ticks were collected from the same hosts; detection rates could therefore not be compared between sampling years or at the host level. Moreover, because multiple ticks were collected from the same host, individual samples are not strictly independent; the pairwise co-detection associations were not statistically significant (bootstrap 95% confidence intervals included zero), and thus these estimates should be interpreted as descriptive measures for this tick sample set rather than as evidence of biological co-infections within ticks. Furthermore, all examined ticks were engorged, and PCR positivity in whole-tick extracts could not distinguish pathogen DNA associated with the ticks from DNA originating from residual host blood. Consequently, this study can only confirm the detection of the above pathogen DNA in tick samples and cannot determine whether *H. marginatum* is actually infected with or capable of transmitting these pathogens. Future studies based on unfed tick specimens, or on pathogen detection in tick salivary glands, are therefore necessary to confirm genuine infection of the ticks. Further validation through pathogen proliferation assays in ticks and animal transmission experiments is warranted to clarify the vector competence of *H. marginatum*. Nevertheless, these findings enrich the molecular surveillance data on tick-borne pathogens associated with *H. marginatum* in Qinghai Province and provide a preliminary scientific basis for the monitoring and control of tick-borne diseases in the local area.

## Figures and Tables

**Table 1:** Primer sequences and amplification conditions used in PCR.

Target	Primer Name		Sequence (5′–3′)	Product Length	Extended Duration	Annealing Temperature	Reference
Tick *16S* rRNA	*16S* rRNA-F		CTGCTCAATGATTTTTTAAATTGCTGTGG	450 bp	45 s	55 °C	[14]
	*16S* rRNA-R		CCGGTCTGAACTCAGATCAAGT				
Tick *cox1*	LCO1490		GGTCAACAAATCATAAAGATATTGG	709 bp	1 min	56 °C	[15]
	HCO2198		TAAACTTCAGGGTGACCAAAAAATCA				
*Theileria* spp.	Primary	Piro1-s	CTTGACGGTAGGGTATTGGC	1400 bp	1 min 30 s	55 °C	[16]
		Piro3-AS	CCTTCCTTTAAGTGATAAGGTTCAC				
	Secondary	PIRO-A	AATACCCAATCCTGACACAGGG	430 bp	45 s	55 °C	
		PIRO-B	TTAAATACGAATGCCCCCAAC				
*T. luwenshuni*	P3		CATCTTCTTTTTGATGAGTTGA	103 bp	20 s	55 °C	[17]
	P4		AAAGCCTGCTTTGAGCACTCTA				
*T. uilenbergi*	YF		CCTCGACGGGCGTTTGCGCCGC	803 bp	1 min	55 °C	[18]
	YR		AACGCATCAGTGTAGCGCGCGT				
*Anaplasma* spp.	Primary	gro607F	GAAGATGCWGTWGGWTGTACKGC	734 bp	50 s	57 °C	[19]
		gro1249 R	AGMGCTTCWCCTTCWACRTCYTC				
	Secondary	gro677F	ATTACTCAGAGTGCTTCTCARTG	364 bp	30 s	53 °C	
		gro1121 R	TGCATACCRTCAGTYTTTTCAAC				

**Table 2:** Centered Jaccard co-occurrence indices, bootstrap 95% confidence intervals, and Fisher’s exact test results for pairwise pathogen co-detection in tick samples.

Pathogen Pair	Centered Jaccard Index	Bootstrap 95% CI	Fisher’s Exact Test *p*-Value
*T. ovis* & *T*. *luwenshuni*	0.085	−0.032 to 0.263	0.139
*A*. *ovis* & *T*. *luwenshuni*	0.069	−0.042 to 0.238	0.209

## Data Availability

The original contributions presented in this study are included in the article. Further inquiries can be directed to the corresponding authors.

## Abbreviations

The following abbreviations are used in this manuscript:

*Hyalomma marginatum*	*H. marginatum*
*Theileria ovis*	*T. ovis*
*Theileria luwenshuni*	*T. luwenshuni*
*Theileria uilenbergi*	*T. uilenbergi*
*Anaplasma ovis*	*A. ovis*
CCHFV	Crimean-Congo hemorrhagic fever virus
